# VEGF and the VEGF_73-101_ Fragment Prevent MPP^+^ Induced Mitochondrial Dysfunction in a Cell Model of Parkinson’s Disease

**DOI:** 10.1007/s12035-025-05213-9

**Published:** 2025-07-31

**Authors:** Stefania Zimbone, Giuseppe Battiato, Stefano Conti Nibali, Irina Naletova, Noemi Anna Pesce, Vito De Pinto, Angela Messina, Andrea Magrì, Marianna Flora Tomasello, Giulia Grasso

**Affiliations:** 1 Institute of Crystallography, National Council of Research, Catania Unit, Catania, Italy; 2 Department of Biomedical and Biotechnological Sciences, University of Catania, Catania, Italy; 3 Department of Biological, Geological and Environmental Sciences, University of Catania, Catania, Italy; 4 MitoBiotech S.R.L, Catania, Italy

**Keywords:** VEGF, Mitochondria, Oxidative phosphorylation (OXPHOS), Parkinson’s disease (PD), Neurotrophic, Peptide, MPP^+^

## Abstract

Vascular endothelial growth factor (VEGF) is best known for its role in angiogenesis, but emerging evidence suggests neurotrophic and neuroprotective functions most likely connected to energy homeostasis pathways. Using high-resolution respirometry, we investigated the role of VEGF in optimizing mitochondrial oxidative phosphorylation efficiency in neuronal-like cells. Our findings indicate that VEGF enhances coupling efficiency for ATP synthesis by reducing the uncoupled respiration, a process involved in mitochondrial dysfunction and neurodegeneration, as observed in Parkinson’s disease (PD). To investigate the VEGF relevance in PD, we exposed differentiated SH-SY5Y cells to MPP^+^, a toxin inducing a PD-like phenotype. Both VEGF and a mimic peptide (VEGF_73-101_) reduced MPP^+^-induced uncoupled respiration, mitochondrial membrane potential (Δψm) collapse, and cell death. These effects were associated with Erk signaling activation, upregulation of PGC-1α genes expression and increased mitochondrial DNA. Our study underscores VEGF’s multifaceted role in neuronal resilience beyond angiogenesis. Furthermore, these findings suggest that the VEGF_73-101_ fragment may retain key neuroprotective and neurotrophic properties supporting further exploration of its therapeutic potential.

**Supplementary Information:**

The online version contains supplementary material available at 10.1007/s12035-025-05213-9.

## Introduction

In mammals, the vascular endothelial growth factor (VEGF) family plays key roles in angiogenesis. Among its members, VEGF-A (and specially the VEGF-165 splicing variant used in this study) [1–3], is the most active, exerting its effects primarily via VEGFR-2, activating numerous signaling pathways [4–6]. For the sake of clarity, we will hereafter use “VEGF” to refer specifically to the human VEGF-A isoform. In the nervous system, Neuropilins (NRP1 and NRP2) enhance VEGF activity when bound to VEGFR-2 [3, 7–10].

Mounting evidence indicates that, beyond vascular regeneration, VEGF is involved in neuronal survival protecting neurons against oxidative stress, excitotoxicity, and ischemia [11–17]. Notably, VEGF overexpression or administration rescues dopaminergic (DA) neurons in Parkinson’s disease (PD) models, supporting its potential as a treatment for neurodegenerative diseases [18–24]. Therefore, VEGF is gaining interest for its neurotrophic and neuroprotective properties [8, 12, 25–27]. However, whether VEGF’s neuroprotection involves mitochondrial function, a key factor in PD, remains unclear.

PD, the second most common age-related neurological disorder, is mainly characterized by the death of DA neurons in the substantia nigra, mitochondrial dysfunction, and α-synuclein aggregation [28, 29]. Mitochondrial impairment, including altered biogenesis, dynamics, and mitophagy, imbalanced reactive oxygen species (ROS), and complex I inhibition, is central in PD pathogenesis. The involvement of mitochondria in PD is further supported by: (1) PD-linked mitochondrial targeted toxins (MPTP, rotenone); (2) mutations in mitochondrial-related genes (PARK2, LRRK2, PINK1); and (3) mitochondrial dysfunction in PD patient brains [30–35].

Considering that VEGF supports DA neurons in various PD models [18, 19, 21, 23, 24] and that it enhances mitochondrial function in other neuropathological settings [36], we hypothesized it might mitigate MPP^+^-induced respiratory dysfunction in a PD context [36]. Here, using differentiated SH-SY5Y cells (a widely recognized PD model [37, 38]), we investigated VEGF’s effects on mitochondrial oxidative phosphorylation (OXPHOS) in physiological and pathological (MPP^+^) conditions by using high-resolution respirometry (HRR) [39, 40]. To further investigate the structure–function relationship, and potentially identify clues for the future therapeutic development, we also examined the activity of a VEGF-derived peptide. This peptide, spanning residues 73–101 [41, 42] of the VEGF sequence, is located at the β5–β6 loop, and includes key sites involved in VEGF-VEGFR-2 binding. Our findings demonstrate that both VEGF and the 73–101 fragment restore mitochondrial respiration efficiency and upregulate PGC-1α-related pathways, increasing mtDNA content suggesting VEGF’s neuroprotection may influence mitochondrial homeostasis. These results highlight VEGF’s role in linking angiogenesis, metabolism, and neuroprotection, warranting further exploration of VEGF-derived peptides for PD treatments.

## Materials and Methods

### Cell Culture and Treatments

Human neuroblastoma cells (SH-SY5Y) were cultivated in Dulbecco’s minimum essential medium F12 (DMEM F12, Gibco), supplemented with 10% v/v fetal bovine serum (FBS) and 100 U/mL of penicillin/streptomycin. The SH-SY5Y cells were grown at 37 °C under 5% CO_2_ atmosphere and used prior to passage 20. For differentiation, we set up a 7-day-long paradigm by seeding SH-SY5Y cells in 1% FBS DMEM F12 supplemented with 5 µM all-trans-retinoic acid (RA) (Sigma). The medium was refreshed every 48 h, and FBS was halved to 0.5%. Recombinant human VEGF 165 (PeproTech, 29 Margravine Road London, UK), hereafter called VEGF, and the designed VEGF_73-101_ peptide (Ac-ESNITMQIMRIKPHQGQHIGEMSFLQHMK-NH_2_, Genecust Europe, Dudelange-Luxembourg), were added to cultures 24 h after medium change without removing it, so that cells did not receive any other factor at the time of treatment. Incubation with VEGF or the peptide lasted 24 h for all parameters assessed and 15 min for western blot experiments. A concentration of 10 ng/ml (0.26 nM) of VEGF was used in accordance with the previous literature reports [27, 43]; a concentration of 5 µM of VEGF_73-101_ was determined by a preliminary dose–response curve (Supplementary Fig. 1). When required, 0.8 mM MPP^+^ was added after 1 h of VEGF/peptide exposure without removing them and kept for 24 h.

### High-Resolution Respirometry

**Fig. 1:**
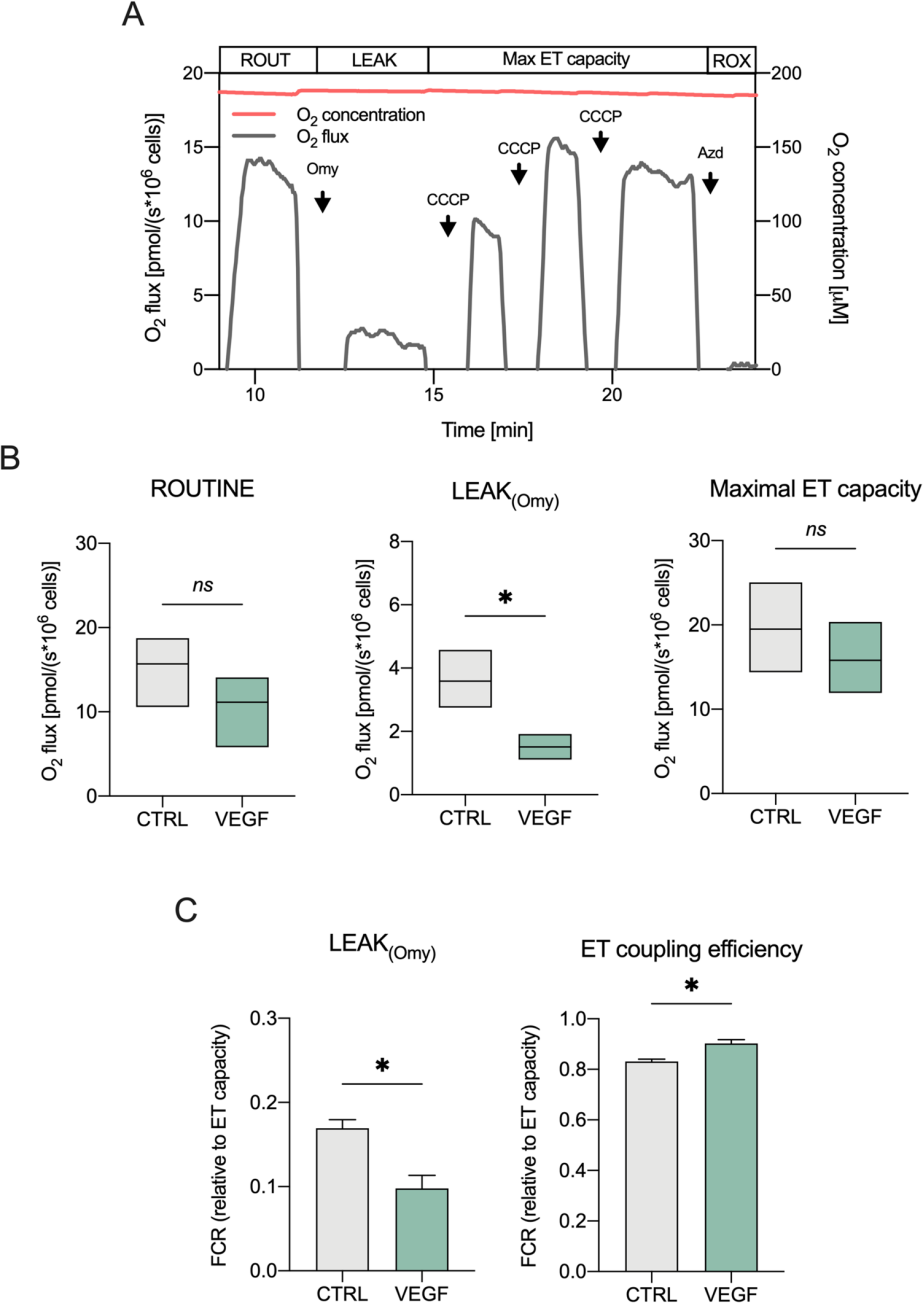
VEGF decreases LEAK respiration in intact differentiated SH-SY5Y cells. **A** Representative curve of mitochondrial respiratory profile of SH-SY5Y along with the SUIT protocol here used for intact cells. Omy, oligomycin; CCCP, carbonyl cyanide 3-chlorophenylhydrazone; Azd, sodium azide. **B**, **C** The oxygen consumption rates relative to ROUTINE, LEAK, and maximal ET capacity (**B**), and the FCRs relative to the LEAK states and the ET coupling efficiency (**C**) in cells exposed for 24 h to 10 ng/ml (0.26 nM) VEGF. Untreated cells were used as control. Data are expressed as means (min to max) (**B**) or means ± SEM (**C**) of *n* = 3 independent experiments and analyzed by unpaired *t* test with *p < 0.05; ns, not significant

The respiratory profile of differentiated SH-SY5Y, exposed to various treatments, was investigated by HRR using the two-chamber system O2k-FluoRespirometer (Oroboros Instruments). Respiratory states were determined by using two specific, alternative substrate-uncoupler-inhibitor titration (SUIT) protocols for both intact and permeabilized cells. In intact cells (Fig. 1A), the basal oxygen consumption (ROUTINE state) was first measured. Then, the uncoupled respiration or LEAK state was achieved by the addition of 1 μM oligomycin in the cuvette (*L*_Omy_). Maximal capacity of electron transport (ET) system (maximal ET capacity) was determined by titration with the uncoupler carbonyl cyanide 3-chlorophenylhydrazone (CCCP, 0.5 μM). Finally, the residual oxygen consumption (ROX state) was measured by the addition of 100 mM sodium azide.

**Fig. 2:**
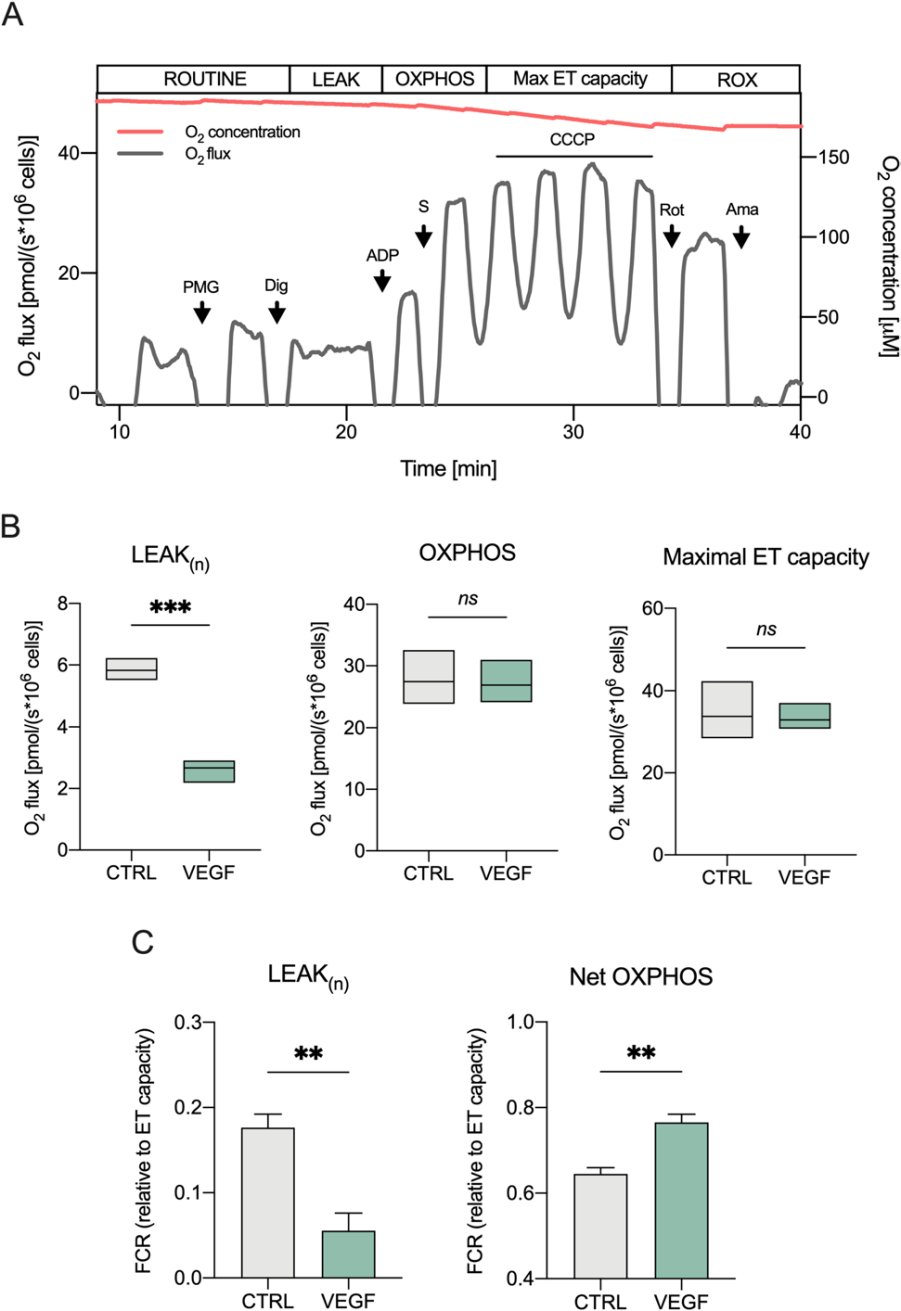
VEGF increases ADP phosphorylation in permeabilized, differentiated SH-SY5Y cells. **A** Representative curve of mitochondrial respiratory profile of SH-SY5Y along with the SUIT protocol adapted for permeabilized cells. P, pyruvate; M, malate; G, glutamate; Dig, digitonin; S, succinate; CCCP, carbonyl cyanide 3-chlorophenylhydrazone; Rot, rotenone; Ama, antimycin. **B, C** The oxygen consumption rates relative to LEAK, OXPHOS, and maximal ET capacity (**B**), and the FCRs relative to the LEAK states and the net OXPHOS (**C**) in cells exposed for 24 h to 10 ng/ml (0.26 nM) VEGF. Untreated cells were used as control. Data are expressed as means (min to max) (**B**) or means ± SEM (**C**) of *n* = 3 independent experiments and analyzed by unpaired *t* test with **p < 0.01, ***p < 0.001; ns, not significant

Alternatively, plasma membranes were permeabilized with 4 μM digitonin (Fig. 2A). The substrates pyruvate (5 mM), glutamate (10 mM), and malate (2 mM) were added to measure the LEAK state in the presence of NADH-linked substrates but not adenylates (*L*_*n*_). Next, oxygen flow related to oxidative phosphorylation (OXPHOS) was achieved with the supplementation of succinate (10 mM) and ADP (2.5 mM). Finally, the maximal ET capacity was determined by titration with CCCP (0.5 μM) while the ROX state with the addition of rotenone (2 μM) and antimycin (2.5 μM). All substrates were purchased by Sigma Aldrich (St. Louis, MO, USA). All experiments were carried out in MiR05 (Oroboros Instruments) at 37 °C under constant stirring. At least three independent experiments were performed for each condition tested.

### Analysis of Respiratory States

Instrumental and chemical background fluxes were calibrated as a function of the oxygen concentration using DatLab software (v7.4.0.1, Oroboros Instruments). Oxygen consumption rates corresponding to ROUTINE (*R*), LEAK (*L*_Omy_, *L*_n_), OXPHOS (*P*), and maximal ET capacity (*E*) were corrected for the ROX and expressed as pmol/s per million cells or as flux control ratios (FCRs) relative to the maximal ET capacity. As previously detailed [44], FCRs for the LEAK states were calculated as *L*/*E*; the ET coupling efficiency was calculated as (*E*-*L*)/*E*; the net OXPHOS was calculated as (*P*-*L*)/E; the R-Reserve was calculated as (*R*-*L*)/*E*.

### Protein Lysate Preparation and Western Blotting Analysis

SH-SY5Y cells were seeded and differentiated in 12-well plates at a density of 1.6 × 10^5^ cells/cm^2^ and exposed to VEGF or VEGF_73-101_ for 15 min to analyze Erk1/2 phosphorylation. After treatments, cells were lysed in RIPA buffer (R0278, Sigma Aldrich) containing a halt protease and phosphatase inhibitor single-use cocktail for 30 min and then centrifuged at 14,000 × *g* for 20 min. Total protein amount was determined by Bradford’s method (Protein Assay Dye Reagent Concentrate, BioRad, Hercules, CA, USA).

For Western blot analysis, equal amounts of proteins were separated by 4–12% tris–glycine gels (Bio-Rad, Hercules, CA, USA) and transferred onto nitrocellulose membranes. Phospho-Erk1/2, VDAC1/3 and COX IV were detected with specific primary antibodies (cat. n. 4370, Cell Signaling; cat. n. ab14734, Abcam; cat. n. 4850, Cell Signaling) by incubation overnight at 4 °C. The Erk1/2 phosphorylation values were normalized to Erk1/2 expression level (cat. n. 9107, Cell Signaling) as indicated. Actin (cat. n. A3853, Sigma-Aldrich) or tubulin (cat. n. 2144, Cell Signaling) were used as a loading control to assess equal sample loading. The appropriate infrared-dye labeled secondary antibodies were used to detect primary antibodies. Membranes were scanned using the Odyssey Infrared Imaging System (LI-COR Biosciences, Lincoln, NE, USA), and quantitative densitometric analysis was performed using ImageJ software. Data are representative of three/four independent experiments with at least two repeats for each condition.

### Biolayer Interferometry Measurements

The kinetic of VEGFR-2 binding to VEGF or VEGF_73-101_ was determined by biolayer interferometry (BLI) using the Octet N1 instrument and evaluated with steady state analysis. BLI technology is an optical system useful for measuring interactions between peptides, proteins, small molecules, nucleic acids, and/or lipids in real time. Experiments were performed in high-salt phosphate buffer PBS with 0.02% Tween20 and 0.1% BSA (KB Buffer, Sartorius, Sartorius Italy S.r.l.) on an Octect N1 System (Sartorius, Sartorius Italy S.r.l.) operating at 25 °C. VEGFR-2 (VEGFR-2-Fc human VEGFR-2/KDR protein (domain 1&2&3, Fc Tag) Sino Biological) 25 nM was loaded onto Protein A Biosensor (ProA, Sartorius, Sartorius Italy S.r.l.) for 800 s in tube, to saturate the sensor. The optimal ligand concentration (25 nM) was properly chosen in the 25–400 nM range. Affinity constant (*K*_*D*_) values were estimated by fitting the response intensity (nm) as a function of analyte concentration (nM or mM). The interaction of the KB buffer with biosensor after VEGFR-2 loading was chosen as control. Binding assays were independently repeated twice. Error bars indicate the standard error of the mean (SEM) (see Supplementary Information for further details).

### Cell Viability Assay

Cells viability was determined using the standard MTT (3-(4,5-dimethylthiazol-2-yl)−2,5-diphenyltetrazolium bromide) assay. SH-SY5Y seeded and differentiated in 96-well plates at a density of 1.5 × 10^4^ cells/cm^2^, were treated with VEGF/peptide and/or MPP^+^ for 24 h, and then incubated, at 37 °C, for 3 h with 5 mg/mL (12 mM) of MTT (stock solution). After incubation, the supernatant was removed; the purple formazan crystals were resuspended in 200 μL of DMSO and gently shaken for 2 min until complete dissolution. Absorbance was measured at 570 nm using the Varioskan Microplate reader (Thermo Fisher). Experiments were performed at least in triplicate and independently repeated three times.

### Cytotoxicity Assay

Cytotoxicity was assessed in real-time using the Sartorius IncuCyte® Cytotox Red Reagent, a membrane-impermeable DNA-binding dye that exhibits minimal fluorescence in healthy cells but undergoes a 100–1000-fold increase upon nuclear entry in compromised cells. Briefly, cells were seeded and differentiated in 96-well plates at the same density used for MTT assay. Prior to treatment, the Cytotox Red dye was added to each well at a final concentration of 250 nM. Treated plates were then transferred to the IncuCyte® Live-Cell Analysis System, where fluorescence (ex/em: ~ 560/615 nm) was acquired at 0, 24, and 48 h. Fluorescence was quantified using the IncuCyte® Basic Analyzer Module, with background subtraction (surface fit method). Cytotoxicity was expressed as red integrated intensity (object average)/phase area confluence, normalized to 0d0h0m. Data represent mean ± SEM of triplicates, with untreated cells as negative control.

#### Analysis of Mitochondrial Membrane Potential and Mitochondrial Mass

ΔΨm was measured using tetramethyl-rhodamine methyl ester (TMRM). TMRM accumulates in active mitochondria due to its positive charge whereby the reduction of ΔΨm leads to the release of TMRM. After treatments, adherent cells seeded in 24-well plates (8 × 10^4^ cells/cm^2^) were washed with PBS and then incubated for 30 min at 37 °C with Krebs Ringer Buffered Saline (130 mM NaCl, 3.6 mM KCl, 10 mM HEPES, 2 mM NaHCO_3_, 0.5 mM NaH_2_PO_4_, 0.5 mM MgCl_2_, 1.5 mM CaCl_2_, 4.5 g/l glucose, pH 7.42) supplemented with 200 nM TMRM and 20 μM Verapamil (a multi drug-resistant pump inhibitor, Sigma). Cells were then detached by short treatment with trypsin–EDTA, re-suspended in the above described buffer, supplemented with 1% FBS to neutralize the trypsin and immediately analyzed on a CyFlow® ML flow cytometer (Partec) in FL3 log mode. Only viable cells, detected by reading the scattering (indicated as FSC and SSC), were considered for our analysis. The threshold physiological value of ΔΨm was estimated by the negative control obtained by cells exposed to 10 μM of the uncoupling agent FCCP [45]. Mitochondrial mass was evaluated by measuring the fluorescence of MitoTracker Green (ThermoFisher) by flow cytometry. Adherent cells were loaded for 20 min with 200 nM of MitoTracker Green according to the manufacturer instructions. Cells were then collected and analyzed on a CyFlow® ML flow cytometer (Partec) on the FL1 log mode (490/516 nm). Only viable cells, identified by forward and side scatter (FSC and SSC), were included in our analysis. For MitoTracker Green, the ratio between the average green fluorescence of the control and our sample was calculated.

### Flow Cytometry

20,000 cells per sample were analyzed using a CyFlow® ML flow cytometer (Partec) system equipped with three laser sources and 10 optical parameters with dedicated filter setting and a high numerical aperture microscope objective (50 × NA 0.82) for the detection of different scatter and fluorescence signals. The cells were excited by an air-cooled argon 488-nm laser, and then, the signal from TMRM and Mitotracker Green were read on FL3 and FL1 detectors respectively. Data obtained were acquired, gated, compensated, and analyzed using the FlowMax software (Partec) and the FCS Express 4 software (DeNovo). Data reported are representative of three to four sets of independent experiments for TMRM, or two independent experiments for Mitotracker Green, each performed in duplicate and based on 20,000 events for each group.

### Real-Time PCR

Quantitative real-time PCR was used to analyze the expression level of PCG-1α, TFAM and NRF-1. Total RNA was extracted using TRIzol® Reagent (ThermoFisher Scientific) according to manufacturer’s instructions. RNA concentration and purity were measured by a spectrophotometer, and 1 μg was used to synthesize cDNA by QuantiTect Reverse Transcription kit (Qiagen). For each experiment, three independent real-time PCR were performed in triplicate using the QuantiTec SYBR Green PCR Kit (QIAGEN). Analysis was performed in the Mastercycler ep-realplex (Eppendorf) in 96-well plates. Thermocycling program consisted in a first activation at 95 °C for 15 min, followed by 40 cycles at 95 °C for 15 s, annealing at 57 °C for 15 s, extension at 68 °C for 15 s and a final step at 72 °C for 10 min. Analysis of relative expression level was performed using the housekeeping β-actin gene as internal calibrator by the ΔΔCt method [46]. Data are representative of three independent experiments performed in triplicate and were statistically analyzed by one-way ANOVA followed by Dunnett’s multiple comparisons test. In Table 1, the pairs of primers used for real-time PCR.

**Table 1:** Primers used for mitochondria DNA analysis

Primer	Sequence
Fw PCG-1α	5’- GATGCGCTGACAGATGGAGA-3’
Rev PCG-1α	5’- TAGCTGAGTGTTGGCTGGTG-3’
Fw TFAM	5’- CGCAGGAAAAGCTGAAGACT-3’
Rev TFAM	5’- TGTGCGACGTAGAAGATCCT −3’
Fw NRF-1	5’- AGTGGCAGCTTCTCAGGAC −3’
Rev NRF-1	5’- ACTCCAGTAAGTGCTCCGAC-3’
Fw β-actin	5’- ACACTGTGCCCATCTACGAG-3’
Rev β-actin	5’- AATGTCACGCATTTCCC-3’
Fw mtDNACOX II	5’- GTACTCCCGATTGAAGCCCC-3’
Rev mtDNACOX II	5’- ACCGTAGTATACCCCCGGTC-3’
Fw nDNA18S	5’- TAGAGGGACAAGTGGCGTTC-3’
Rev nDNA18S	5’- CGCTGAGCCAGTCAGTGT-3’

### Mitochondrial DNA Analysis

To determinate the relative amount of mitochondrial DNA, total DNA was extracted as described in [47]. Briefly, total DNA (∼20 ng) was used as a template in real-time PCR with primers for the COXII gene in mitochondrial DNA and nuclear gene for 18S. Analysis of mtDNA/nDNA ratio was calculated by following the classical ΔΔCt method. Data are representative of three independent experiments performed in triplicate and were statistically analyzed by one-way ANOVA. In Table 1, the pair of primers used for mitochondria DNA analysis.

### Statistical Analysis

Data are presented as mean ± SEM. Dependent variables were analyzed by either unpaired *t* test-two tailed or one-way/two-way ANOVA followed by Tukey/Dunnett’s multiple comparisons test, depending on the data analyzed, using GraphPad Prism version 9.0.0 (GraphPad Software, San Diego, CA, USA). A value of *p* < 0.05 was considered significant. In particular, one asterisk (*) indicates a *p* < 0.05, two asterisks (**) indicate a *p* < 0.01, three asterisks (***) indicates a *p* < 0.001, and four asterisks (****) indicates a p < 0.0001.

## Results

### VEGF Enhances Mitochondrial Coupling by Restricting Non-phosphorylating Respiration

Previous studies have highlighted the neuroprotective effects of VEGF in PD models; however, poor knowledge is available with respect to the connection between VEGF and mitochondrial physiology in neuronal cells. Therefore, we initially assessed whether VEGF could impact the mitochondrial energy transduction system in physiological condition. For this purpose, HRR measurements were carried out in differentiated SH-SY5Y cells [39]. This technique allows the measurement of mitochondrial respiration in different respiratory states, here displayed as both oxygen flux or flux control ratios (FCRs), the last showing how much each state contributes to reaching the maximal ET capacity.

Differentiated SH-SY5Y previously exposed to 24 h of 10 ng/ml (0.26 nM) VEGF were first assessed using a HRR protocol which evaluates oxygen consumption in intact cells. Figure 1A reports a representative curve of oxygen consumption supported by endogenous substrates (ROUTINE) registered in untreated differentiated SH-SY5Y cells (control). The uncoupled respiration or LEAK (*L*_Omy_), corresponding to the dissipative component of respiration, was measured by adding oligomycin, which specifically inhibits the ATP synthase. Then, the maximal ET capacity was determined by titration with the uncoupler CCCP. Finally, ROX was calculated inhibiting ETC by sodium azide. The oxygen flows relative to the main respiratory states are reported in Fig. 1B. While no significant differences were observed in the ROUTINE or maximal ET capacity, oxygen flow was significantly reduced in the non-phosphorylating LEAK respiration (*p* = 0.023 vs. control, *n* = 3). Interestingly, a similar trend emerged from the FCR analysis of LEAK, which represents the contribution of uncoupled respiration to the maximal ET capacity. As shown in Fig. 1C, the LEAK was significantly reduced by about 42% upon VEGF treatment (*p* = 0.018 vs. control, *n* = 3). These data suggest that VEGF decreases the proton and/or electron leakage.

Next, we analyzed the ET coupling efficiency, which represents the amount of oxygen used for ADP phosphorylation compared to the total oxygen consumed, a useful parameter to evaluate both physiological and pathological uncoupling. The total oxygen consumption is calculated, independently of the rate at which ATP is made and is tested in experiments using CCCP [48]. As reported, VEGF promoted a slight but significant increase in the coupling efficiency of about 7% (*p* = 0.018 vs. control, *n* = 3, Fig. 1C), confirming the positive effect exerted by VEGF.

### VEGF Improves Bioenergetic Efficiency by Optimizing the ATP-Related Oxygen Flux

To validate these findings, we next performed additional HRR measurements using an alternative protocol involving the mild permeabilization of the plasma membrane of studied cells. This approach allows (i) the evaluation of the LEAK respiration in the absence of adenylates but using externally added substrates to stimulate the OXPHOS state (*L*_*n*_), (ii) the analysis of ATP coupled respiration tightly dependent on the phosphorylation system. As shown in the representative curve of Fig. 2A, the LEAK was measured immediately after the plasma membrane permeabilization with digitonin. Later, when combination of pyruvate, malate, glutamate, succinate, and ADP entered the cells, the OXPHOS respiration was assayed.

Again, VEGF treatment significantly reduced oxygen flux in the LEAK state (*p* < 0.001 vs. control, *n* = 3, Fig. 2B), demonstrating reproducibility across complementary experimental methodologies. Notably, no changes were observed in OXPHOS respiration or maximal ET capacity (Fig. 2B). Once more, FCR analysis confirmed a significant reduction in the LEAK respiration (approx. 69% compared to the control, *p* = 0.0097 vs. control, *n* = 3, Fig. 2C). While the total OXPHOS respiration remained substantially unchanged, the fully coupled phosphorylating component, which corresponds to the oxygen flux employed for the ATP production (the so-called net OXPHOS), was significantly increased upon VEGF exposure (+ 19%, *p* = 0.0079 vs. control, *n* = 3, Fig. 2C).

Taken together, HRR data indicate that VEGF improves mitochondrial respiration, by reducing LEAK respiration, thereby increasing ATP production efficiency.

### VEGF Protects from MPP^+^-Induced Mitochondrial Dysfunction

The ability of VEGF to improve mitochondrial coupling efficiency and optimize energy utilization could be pivotal in certain pathological conditions involving energy deficits, such as PD. To evaluate the beneficial effect of VEGF in this context, we exposed differentiated SH-SY5Y to MPP^+^, known to cause an irreversible PD-like syndrome. We have also previously demonstrated that the MPP^+^ alters the respiratory profile of differentiated SH-SY5Y, specifically increasing LEAK respiration [39].

**Fig. 3:**
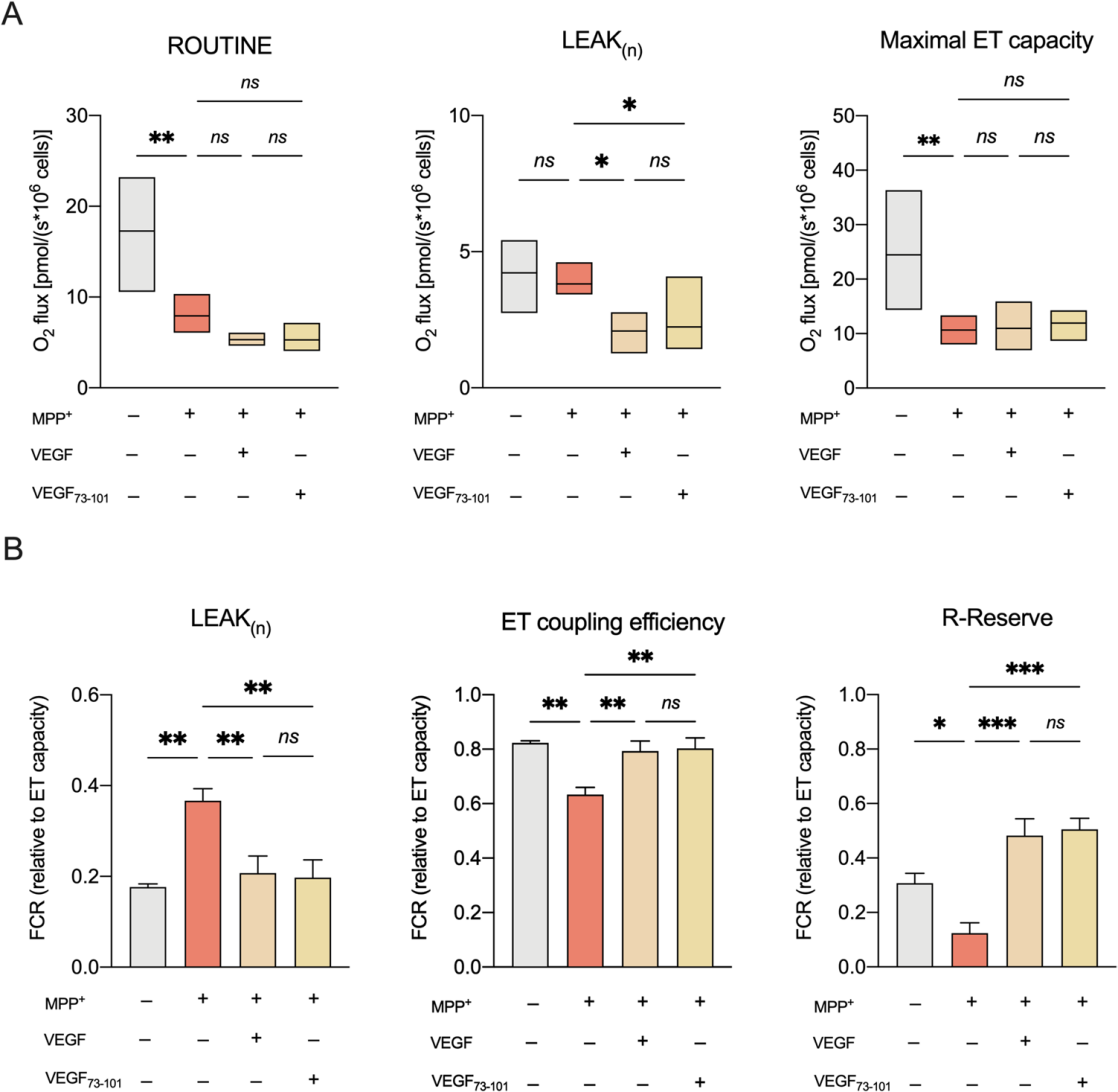
VEGF and its fragment 73–101 recover mitochondrial respiratory profile of differentiated SH-SY5Y cell exposed to MPP^+^. The oxygen consumption rates relative to ROUTINE, LEAK and maximal ET capacity (**A**) and FCRs relative to the LEAK state, ET coupling efficiency and R-Reserve (**B**) of SH-SY5Y cells exposed to MPP^+^, in the presence or not of VEGF and fragment VEGF_73-101_. Untreated cells were used as control. Data are expressed as means (min to max) (**A**) or means ± SEM (**B**) of *n* = 5 independent experiments and analyzed by one-way ANOVA followed by Tukey’s test with *p < 0.05, **p < 0.01, and ***p < 0.001; ns, not significant

HRR measurements were carried out following 24 h exposure to MPP^+^ on differentiated SH-SY5Y cultures previously incubated with VEGF (10 ng/ml/0.26 nM) for 1 h. Confirming our previous findings, MPP^+^ impaired mitochondrial respiration in differentiated SH-SY5Y, specifically decreasing oxygen consumption associated with ROUTINE and maximal ET capacity (*p* = 0.0012 and *p* = 0.0017 vs. control respectively, *n* = 5, Fig. 3A). In particular, the effect of MPP^+^ resulted in a twofold increase in the FCR relative to the LEAK (*p* = 0.002 vs. control, *n* = 5, Fig. 3B), while reducing the coupling efficiency by 19% (*p* = 0.0022 vs. control, *n* = 5, Fig. 3B). This respiratory profile is indicative of a pathological uncoupling [44]. In addition, MPP^+^ treatment reduced the respiratory reserve (R-Reserve) by approximately 60% (*p* < 0.05 vs. control, *n* = 5, Fig. 3B). Notably, R-Reserve represents the additional, potential ATP production through oxidative phosphorylation that can support increased energy demands, acting as a critical mitochondrial reserve under stressful conditions.

Interestingly, VEGF pre-treatment was able to limit the detrimental effect of MPP^+^. As shown in Fig. 3A, VEGF reduced the oxygen consumption relative to the LEAK (*p* = 0.027 vs. MPP^+^ treated cells, *n* = 5), although it did not fully restore oxygen fluxes related to ROUTINE and maximal ET capacity. Of note, FCR analysis revealed that VEGF reduced LEAK respiration, to the control level (*p* < 0.01 vs. MPP^+^ treated cells, *n* = 5) while increasing both coupling efficiency and the R-Reserve, ultimately restoring all parameters to those of the untreated controls (Fig. 3B). Specifically, VEGF increased coupling efficiency roughly by 16% (*p* < 0.01 vs. MPP^+^ treated cells, *n* = 5), while R-Reserve was four times higher than in MPP^+^-treated samples (*p* = 0.0002 vs. MPP^+^-treated cells, *n* = 5).

### The VEGF_73-101_ Peptide recapitulates VEGF’s Neuroprotective Features

To gain deeper insight into the protein region responsible for stimulating mitochondrial respiration and explore the idea of developing new peptide-derived drugs that mimic the effects of VEGF, we investigated the VEGF_73-101_ fragment for its potential to restore MPP⁺-induced mitochondrial dysfunction. This peptide, spanning VEGF’s β5-β6 loop (residues 73–101) with N- and C-terminal blocking groups, contains critical VEGFR-2 interaction sites. Intriguingly, similar to VEGF, 5 µM VEGF_73-101_ reduced the LEAK respiration in untreated cells (Supplementary Fig. 2), extending its protective properties to MPP^+^-treaded cells. Indeed, as shown in Fig. 3, VEGF_73-101_ reduced the MPP^+^-induced LEAK in terms of both oxygen consumption and FCR (*p* = 0.047 and *p* < 0.01 respectively vs. MPP^+^-treated cells, *n* = 5), promoting in turn the recovery of coupling efficiency and R-Reserve to the control levels (*p* < 0.01 vs. MPP^+^-treated cells, *n* = 5). Overall, these data indicate that VEGF_73-101_ behaves similarly to VEGF in preserving mitochondrial bioenergetics upon stress stimuli.

**Fig. 4:**
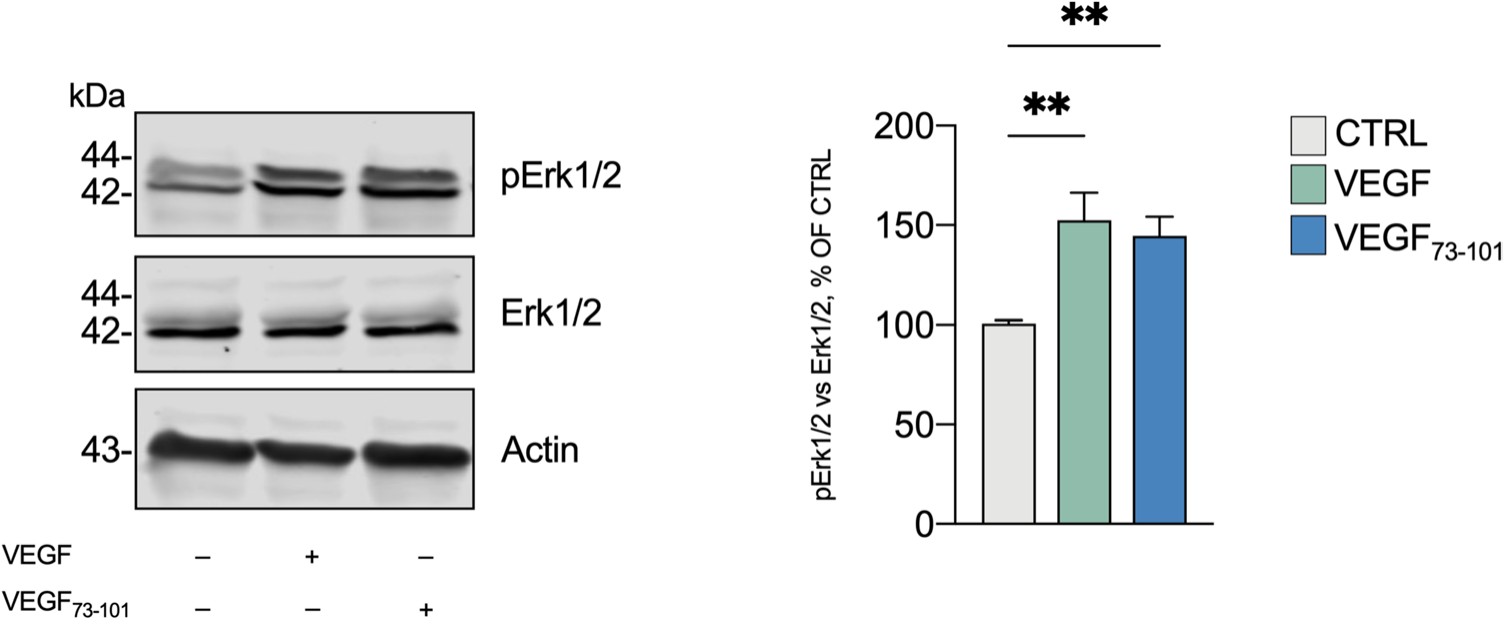
VEGF and VEGF_73-101_modulate Erk1/2 phosphorylation. Differentiated SH-SY5Y cells were treated with VEGF (10 ng/ml/0.26 nM) or VEGF_73-101_ (5 µM) for 15 min. Densitometric analysis and representative Western blotting images of phospho-Erk1/2 in SH-SY5Y cells. The phosphorylation level of Erk1/2 is normalized to total Erk1/2 expression level and expressed as a percentage of control cells. Data are expressed as mean ± SEM of *n* = 4 independent experiments with at least *n* = 2 each. *p ≤ 0.05, **p ≤ 0.01 vs. control untreated cells as a result of the one-way ANOVA

Given that VEGF_73-101_ contains the VEGFR-2’s interaction domain and that it recapitulates the VEGF’s effects on mitochondrial bioenergetics, we hypothesize VEGFR-2-mediated mechanisms. Specifically, we assessed Erk1/2 phosphorylation, a canonical downstream pathways activated by VEGF/VEGFR-2 interaction, well-known for influencing mitochondrial physiology trough multiples mechanisms [49]. Figure 4 shows that 5 µM of either VEGF_73-101_ or VEGF activated Erk1/2 (144% ± 20 vs. 152% ± 31, respectively), supporting the hypothesis of VEGFR-2-mediated mechanisms. The VEGF_73-101_/VEGFR-2 interaction was further validated by using biolayer interferometry (BLI) to quantitatively measure binding kinetics, obtaining direct measurements of association/dissociation rates and equilibrium affinity. Consistent with the values reported in literature [50, 51], VEGF showed high VEGFR-2 affinity binding, with a *K*_*D*_ of 3.5 nM (Supplementary Fig. 3). BLI also revealed that VEGF_73-101_ binds VEGFR-2 with a *K*_*D*_ of roughly 123.8 µM (Supplementary Fig. 3), indicating a VEGFR-2/Erk1/2 involvement.

### VEGF and VEGF_73-101_ Support Mitochondrial Potential to Counteract MPP^+^ Neurotoxicity

**Fig. 5:**
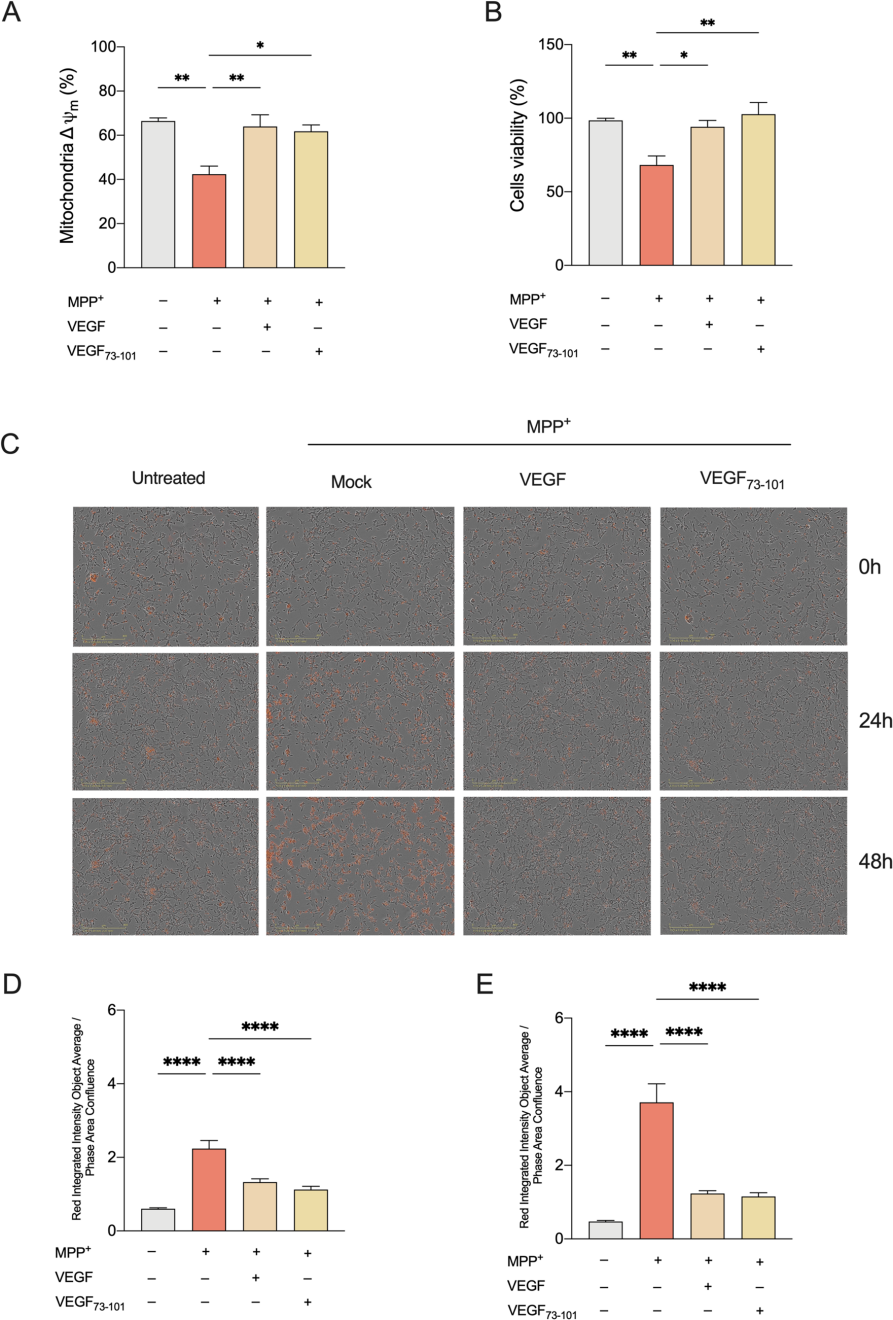
VEGF and peptide VEGF_73-101_ relief MPP^+ ^toxicity. Analysis of mitochondrial membrane potential (**A**) and cell viability (**B**) of differentiated SH-SY5Y cells treated for 24 h with MPP^+^, in the presence or absence of VEGF and VEGF_73-101_. Cytotoxicity assay of differentiated SH-SY5Y cells pretreated or not with VEGF or VEGF_73-101_ peptide for 1 h before being exposed to MPP^+^ for 24 h or 48 h (**C**–**E**). Incucyte images showing Cytotox Red-positive cells taken at 0, 24 and 48 h of run time at 10 × objective **(C)**. Quantification of death cells after 24 h (**D**) and 48 h (**E**) treatment**.** Results are expressed as the ratio of red integrated intensity (object average) to the phase area confluence, normalized to 0d0h0m. Data in (**A**) are expressed as means ± SEM of *n* = 3/4 independent experiments each performed in duplicate. Data in (**B**) are expressed as means ± SEM of *n* = 3 independent experiments performed at least in triplicate. Data in (**D**) and (**E**) represent the mean of ± SEM of triplicates, with untreated cells as negative control. Data were analyzed by one-way ANOVA followed by Tukey’s test with *p < 0.05, **p ≤ 0.01, and ****p < 0.0001

Having shown the ability of VEGF and VEGF_73-101_ to improve mitochondrial coupling efficiency, we next monitored the mitochondrial membrane potential (Δψ_m_) a critical driver of oxidative phosphorylation. According to the literature [52, 53], MPP^+^ treatment induced a substantial decrease in ΔΨm. This effect was significantly attenuated by pretreatment with either VEGF (10 ng/ml; 0.26 nM) or VEGF_73-101_ (5 μM), which maintained ΔΨm at 85% and 78% of control levels, respectively (*p* < 0.01 vs. MPP^+^ alone, Fig. 5A). In contrast, no significant effect on Δψ_m_ was observed when untreated cells were exposed to VEGF or VEGF_73-101_ (Supplementary Fig. 4). These findings further strengthen the evidence that VEGF_73-101_ mimics the protective properties of VEGF in preserving the MPP^+^-induced mitochondrial impairment.

Moving forward, we used MTT assay to assess cell viability in differentiated SH-SY5Y cultures pre-treated for 1 h with 10 ng/ml (0.26 nM) VEGF or 5 µM VEGF_73-101_, before being exposed to MPP^+^ for 24 h. Both VEGF and VEGF_73-101_, almost completely rescued cells from the MPP^+^-induced cell death (Fig. 5B), while neither VEGF-A nor the VEGF_73-101_ affected cell viability or morphology in untreated SH-SY5Y cultures, confirming their lack of intrinsic toxicity at the tested concentrations (Supplementary Fig. 5 and 6). The MTT results were further validated using the IncuCyte Cytotox Red Reagent (Sartorius), a membrane-impermeable dye that selectively enters cells with compromised membrane integrity, thereby acting as a marker of cell death. Images were acquired at 0, 24, and 48 h using the IncuCyte live-cell analysis system (Fig. 5C). As shown in Fig. 5D–E, pretreatment with either VEGF (10 ng/ml; 0.26 nM) or VEGF_73-101_ (5 μM) significantly protected differentiated SH-SY5Y cells from MPP^+^-induced cell death at both 24 h and 48 h timepoints (*p* < 0.0001 vs. MPP^+^ alone; *n* = 3).

### VEGF and the VEGF_73-101_ Fragment Stimulate the PGC-1α Cascade in Differentiated SH-SY5Y Cells

**Fig. 6:**
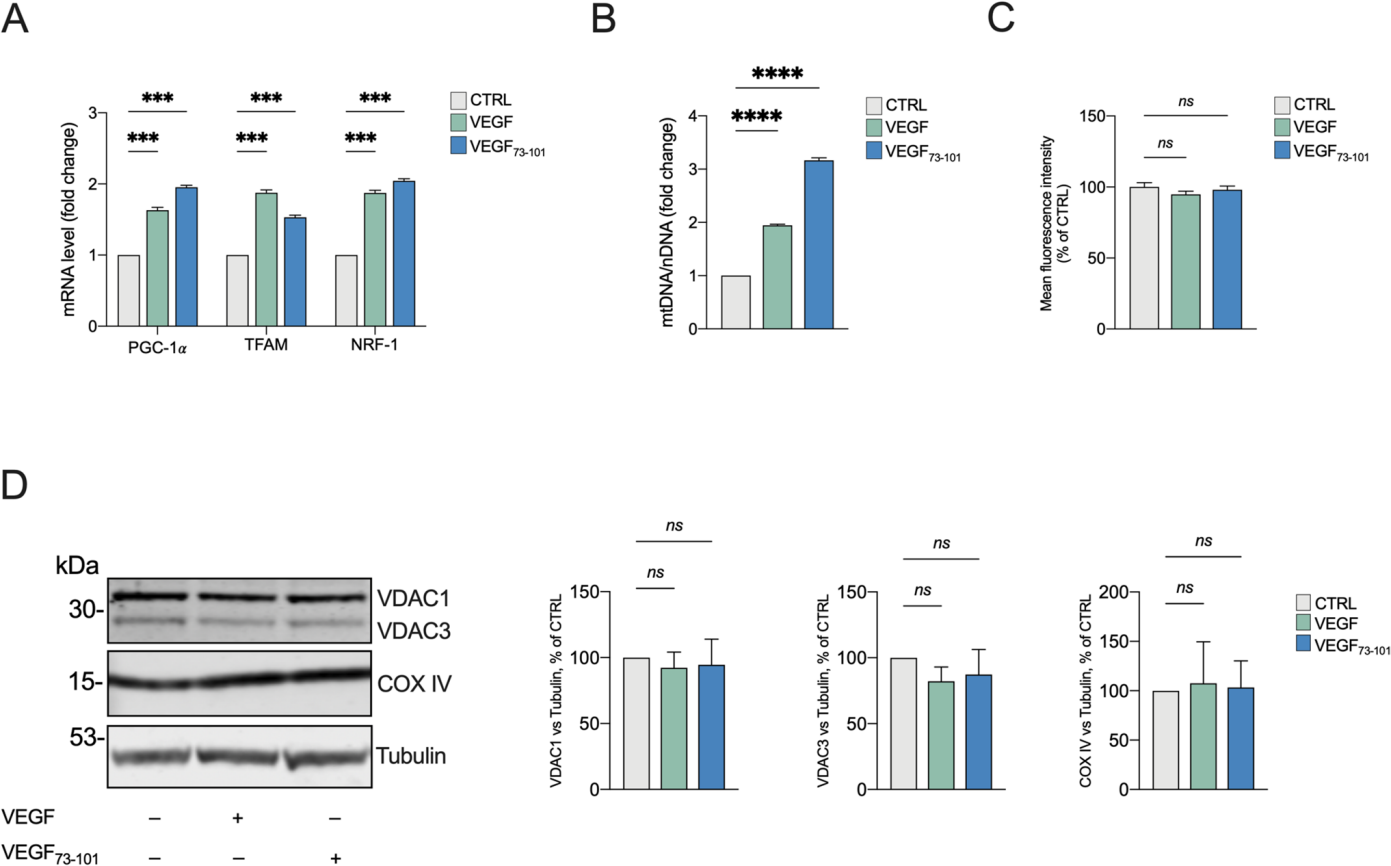
VEGF and VEGF_73-101_ stimulate PGC-1α/TFAM/NRF-1 axis in differentiated SH-SY5Y. **A** Relative quantification of PGC-1α/TFAM/NRF-1 mRNAs in SH-SY5Y cells exposed to VEGF or VEGF_73-101_. β-actin was used as internal loading control. Data are expressed as means ± SEM of *n* = 3 independent experiments each performed in triplicate and compared to untreated cells. Data were analyzed by one-way ANOVA followed by Dunnett’s test with ***p < 0.001. **B** Quantification of mtDNA/nDNA ratio in SH-SY5Y cells exposed to VEGF or VEGF_73-101_. The gene COX II was used as a marker of mtDNA, while the nuclear gene 18S was used for normalization. **C** Measurement of mitochondrial mass using MitoTracker Green in cells exposed to VEGF or VEGF_73-101_. Values reported represent the mean fluorescence intensity of each sample and are expressed as percentage to CTRL (untreated cells = 100%). **D** Representative Western blotting images and densitometric analysis of VDAC1, VDAC3 and COX IV, normalized to tubulin and expressed as percentage of control cells. Data in (**B**) are expressed as means ± SEM of *n* = 3 independent experiments each performed in triplicate and compared to untreated cells. Data are expressed in (**C**) as means ± SEM *n* = 2 independent experiments and in (**D**) as means SEM *n* = 3 independent experiments and compared to control untreated cells. Data were analyzed by one or two-way ANOVA followed by Tuckey’s test with ***p < 0.001 and ****p < 0.0001; ns, not significant

VEGF has been previously reported to stimulate mitochondrial biogenesis in endothelial cells by increasing PGC-1α [54]. PGC-1α interacts with several transcription factors, including the nuclear respiratory factor NRF-1 which promotes the expression of several mitochondrial proteins. Furthermore, NRF-1 regulates the mtDNA transcription by modulating the expression of the mitochondrial transcription factor A (TFAM). Thus, the PGC-1α-NRF-1-TFAM axis works together to support mitochondrial biogenesis and regulate their proper functioning. To investigate whether the bioenergetic improvements demonstrated in our SH-SY5Y model (restored ΔΨm, enhanced respiratory coupling) connect with VEGF’s ability to regulate mitochondrial biogenesis in endothelial cells via PGC-1α [54], we used qPCR to analyze the PGC-1α-NRF-1-TFAM pathway in differentiated SH-SY5Y cells grown for 24 h in the presence of either 10 ng/ml (0.26 nM) VEGF or 5 µM VEGF_73-101_. As shown in Fig. 6A, VEGF and VEGF_73-101_ significantly increased the expression of PGC-1α, NRF-1, and TFAM genes (*p* < 0.001 vs. CTRL, *n* = 3). In line with this, the mtDNA/nDNA ratio was increased by approximately of two or three times in cells exposed for 24 h to respectively VEGF or VEGF_73-101_ (Fig. 6B). To evaluate whether the activation of the PGC-1α-NRF-1-TFAM axis modulates mitochondrial mass, SH-SY5Y cells were loaded with Mitotracker Green, a widely used cell-permeable fluorescent dye that accumulates within mitochondria in live cells, making it a reliable marker for mitochondrial mass quantified by measuring fluorescence in flow cytometry. As shown on Fig. 6C, no significant differences were observed between samples analyzed. Similarly, the expression levels of VDAC1/3 and COXIV, mitochondrial proteins widely used as markers of mitochondrial abundance, remained substantially unchanged across our samples, as demonstrated by Western blot and densitometric analysis (Fig. 6D). Together, these results suggest that the beneficial effects of VEGF and VEGF_73-101_ stem from improved mitochondrial quality rather than increased mitochondrial content.

## Discussion

In this study, we found that VEGF and its 73–101 fragment preserve mitochondrial function in neuronal-like cells by: (i) enhancing bioenergetic efficiency under physiological conditions evidenced by reduced LEAK respiration and increased ATP production; (ii) protecting against MPP⁺-induced toxicity in a PD-like model by sustaining cell viability and mitochondrial function; and (iii) potentially engaging the Erk signaling pathway and upregulating mitochondrial biogenesis regulators, as indicated by pathway analysis.

While VEGF is best known for its angiogenic properties [10], our data are in line with emerging evidence of its direct neuroprotective effects recently reported via VEGFR-2/NP-1 signaling [55, 56]. Notably, previous studies indicated VEGF’s neuroprotection in ischemia [9, 57] and motoneuron disorders [58–61], but our work specifically reveals how its protective properties impact mitochondrial functioning in a PD-relevant context. The results here reported might be relevant for therapeutic strategies targeting metabolic dysfunction in neurodegeneration. However, as our findings are limited to in vitro models, translational potential requires validation in vivo.

On this basis, we used HRR to assess the complete mitochondrial respiratory profile in differentiated SH-SY5Y exposed to VEGF. We found that VEGF lowers the LEAK respiration in terms of both oxygen flux and FCR. This result was reproduced via ATP synthase inhibition using two different experimental approaches: (i) by using oligomycin; and (ii) by removing substrates that exited the cells following plasma membrane permeabilization. Regardless of the protocol used, VEGF optimized fuel exploitation, as further supported by the increase in ATP-linked fluxes (ET coupling efficiency and net OXPHOS).

The LEAK respiration represents the measure of the intrinsic uncoupled respiration due to the physiological leakage of both protons and electrons [48]. However, as it represents the dissipative component of respiration, it is commonly acknowledged that unrestrained LEAK implies impaired mitochondrial coupling efficiency. Therefore, decreasing the LEAK component of respiration might represent a promising strategy to counteract pathological conditions associated with energy failure such as PD [62].

Neurons, with their limited glycolytic capacity [63, 64], must rely on efficient OXPHOS, a process vulnerable to uncoupling-induced heat/ROS in neurodegeneration [62, 65]. Our data show that VEGF improves ETC coupling, therefore reducing these risks. While VEGF’s angiogenic features could indirectly support metabolic demand [9], its direct stabilization of mitochondrial efficiency (demonstrated here) offers a further neuroprotective strategy.

In this work, HRR analysis revealed that both VEGF and the VEGF_73-101_ fragment, containing the VEGFR-2 minimal interaction sites, fully restore mitochondrial coupling efficiency in MPP^+^-treated SH-SY5Y cells, preventing the toxin-induced LEAK respiration increase, Δψm collapse, and cytotoxicity, key hallmarks of PD-related mitochondrial dysfunction.

These findings align with the long-established link between mitochondrial impairment and PD, first highlighted by MPTP/MPP^+^ models [66]. Like MPP^+^, PD-associated mutations (e.g., in PINK1, Parkin) disrupt complex I–driven OXPHOS [67, 68], but our work reveals VEGF’s ability to restore coupling efficiency even after MPP^+^ damage, a mechanism distinct from prior studies focusing on VEGF’s angiogenic or anti-apoptotic roles [10, 55]. Notably, the VEGF_73-101_ peptide recapitulated the full-length protein’s protective effects, suggesting these properties involve the stimulation of VEGFR-2 mediated pathways.

One of the most well-characterized pathways mediated by the VEGF/VEGFR-2 interaction involves Erk, whose phosphorylation in the nervous system is commonly associated with cell survival and neuroprotection [69–71]. In this study, we provide evidence that VEGF_73-101_, similar to VEGF, can modulate Erk1/2 signaling thereby influencing mitochondrial physiology.

This is consistent with studies supporting Erk’s role in the maintenance of mitochondrial integrity, particularly under metabolic stress [49, 72]. In macrophages, for instance, Erk sustains ATP production and Δψm, while its inhibition leads to bioenergetic collapse [73, 74]. Similarly, Erk has been involved in protection against rotenone-induced injury in SH-SY5Y cells [49] and in retinal ganglion cells [75], supporting our observations of VEGF’s mitochondrial-stabilizing effects.

Equally significant was our finding that both VEGF and its 73–101 fragment upregulate PGC-1α/NRF1/TFAM axis, a key regulator of mitochondrial biogenesis, while also increasing the mtDNA/nDNA ratio without altering overall mitochondrial mass. This result is particularly relevant given that proper mtDNA/nuclear genomic coordination is essential for assembling functional respiratory complexes [76], and PD patients frequently exhibit pathogenic mtDNA deletions that impair oxidative phosphorylation and promote ROS generation [77].

**Fig. 7:**
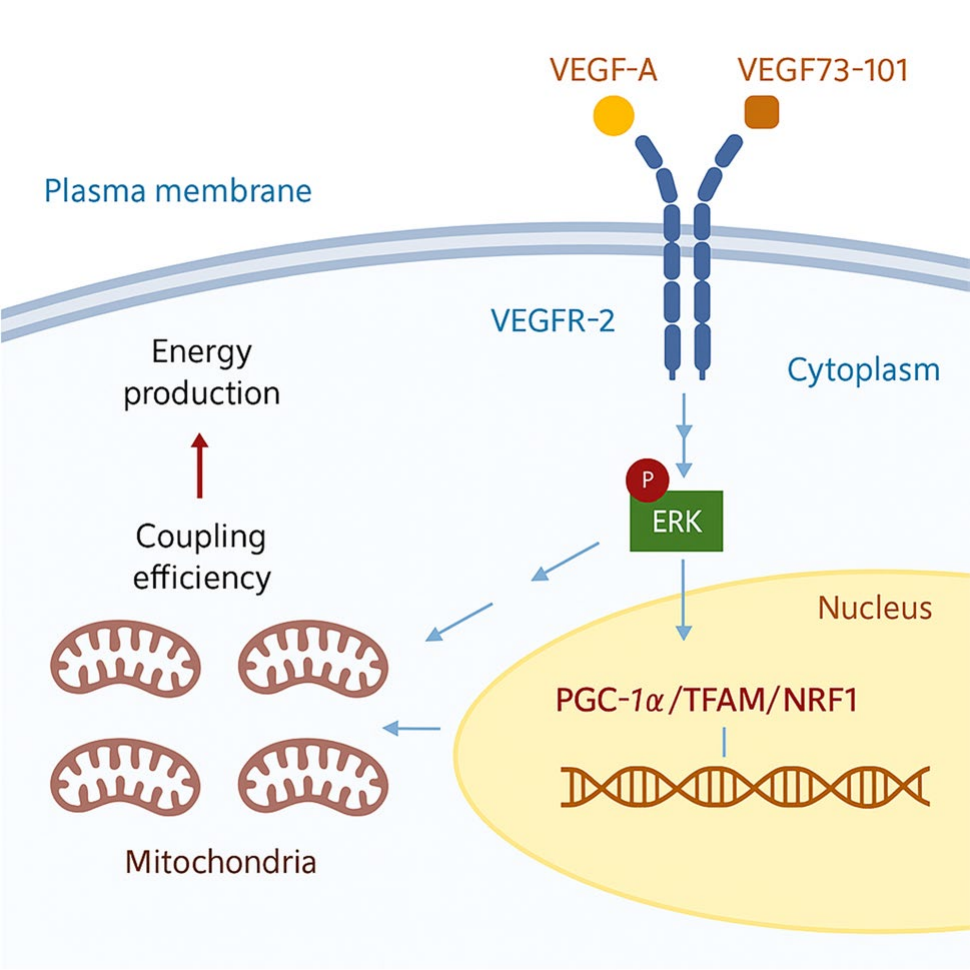
Schematic representation of the model proposed in this work. VEGF might activate Erk-mediated pathways, stimulating the expression of genes involved in the maintenance and/or enhancement of mitochondrial functionality. Picture created by using BioRender software

Our findings position VEGF-A as a key regulator of mitochondrial homeostasis in neuronal-like cells. The observed improvements in respiratory efficiency and neuroprotection in MPP⁺-treated SH-SY5Y cells suggest that VEGF enhances mitochondrial quality rather than quantity, a mechanism that may be particularly relevant for addressing mitochondrial dysfunction in PD and other neurodegenerative conditions. These results extend earlier studies demonstrating VEGF’s role in mitochondrial regulation. For instance, in endothelial cells, VEGF activates the S1P3/Edg3/Akt3 pathway, promoting mitochondrial biogenesis via TOM70 and PGC-1α nuclear translocation [54]. Although the mechanisms in neurons may differ, our data suggest that VEGF similarly supports metabolic adaptation in post-mitotic cells, potentially through Erk-mediated pathways that optimize energy production while limiting ROS generation and thermal stress (Fig. 7). This ability to align metabolic demand with mitochondrial capacity underscores VEGF’s broader role in cellular energetics.

## Conclusions and Study Limitations

This study advances our understanding of mitochondrial involvement in neurodegeneration, specifically positioning VEGF modulation as a strategy for addressing PD. The observed effects on PGC-1α signaling and mtDNA maintenance gain particular relevance given clinical studies linking PGC-1α activation to reduced PD risk [78, 79]. Our work provides mechanistic support for this approach by demonstrating VEGF’s capacity to enhance mitochondrial quality through PGC-1α upregulation, effects which are achieved even by its 73–101 fragment. This dual advance, which elucidates neuroprotective mechanisms while identifying a minimal active sequence, provides both biological insights and practical advantages for drug development. However, several critical questions require further exploration.

Our findings, though compelling, derive from SH-SY5Y cells, a valuable but simplified system that cannot fully replicate human PD pathology. Important next steps include validation in human iPSC-derived neurons and α-synucleinopathy mouse models, along with characterization of the VEGF_73-101_ fragment’s pharmacokinetics, potential off-target effects, the fragment’s behavior in vivo, and its potential synergy with existing therapies. Future studies should also explore the specific mechanisms through which VEGF enhances mitochondrial quality, such as potential effects on mitophagy or fission/fusion dynamics, and might consider monitoring cellular energetics as a complementary diagnostic or prognostic tool. While challenges remain, our findings strengthen the rationale for targeting VEGF pathways in neurodegenerative disorders and provide foundations for subsequent investigations.

## Supplementary Information

Below is the link to the electronic supplementary material.

ESM 1 (DOCX 954 KB)

ESM 2 (PDF 362 KB)

## Data Availability

No datasets were generated or analysed during the current study.
