# Supplementary material for: VEGF and the VEGF73-101 Fragment Prevent MPP+ Induced Mitochondrial Dysfunction in a Cell Model of Parkinson’s Disease
Source: Mol Neurobiol. 2025 Jul 31;62(12):16045–60. doi: 10.1007/s12035-025-05213-9 (PMC12559092; doi:10.1007/s12035-025-05213-9)
Supplement: Supplementary file 1 — (DOCX 954 KB) [file 12035_2025_5213_MOESM1_ESM.docx]

Supplementary Information for:

**VEGF and the VEGF73-101 fragment prevent MPP^+^ induced mitochondrial dysfunction in a cell model of Parkinson's disease.**

Stefania Zimbone^1†^, Giuseppe Battiato^2†^, Stefano Conti Nibali^2^, Irina Naletova^1^, Noemi Anna Pesce^1^, Vito De Pinto^2,4^, Angela Messina^3,4^, Andrea Magrì^3,4^*, Marianna Flora Tomasello^1^* and Giulia Grasso^1^

1 Institute of Crystallography, National Council of Research, Catania Unit, Catania, Italy.

2 Department of Biomedical and Biotechnological Sciences, University of Catania, Italy

3 Department of Biological, Geological and Environmental Sciences, University of Catania, Italy

4 we.MitoBiotech S.R.L., Catania, Italy

†These authors contributed equally to this work and share first authorship

*Correspondence: andrea.magri@unict.it; mariannaflora.tomasello@cnr.it

**Suppl. Fig. 1**

**
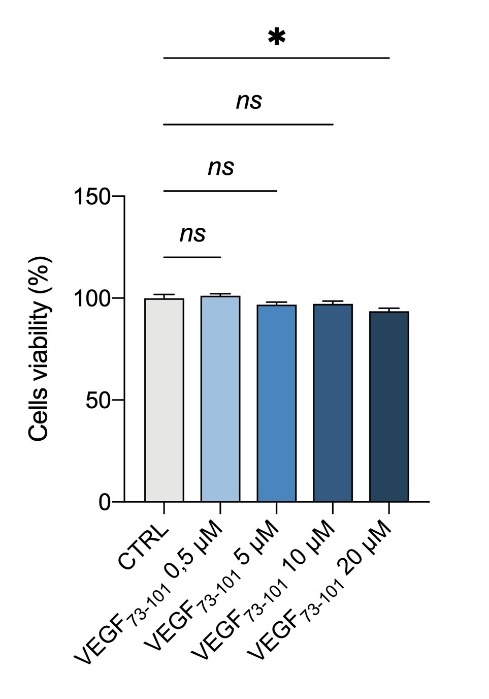
Supplementary Figure 1** Analysis of cell viability by MTT assay of differentiated SH-SY5Y cells treated or not with increasing concentration of VEGF_73-101_ peptide (0,5-20 µM) for 24 h. Data are expressed as means ± SEM of n=3 independent experiments each performed at least in triplicate and analyzed by one-way ANOVA followed by Dunnet test with *p<0.05; ns, not significant.

**
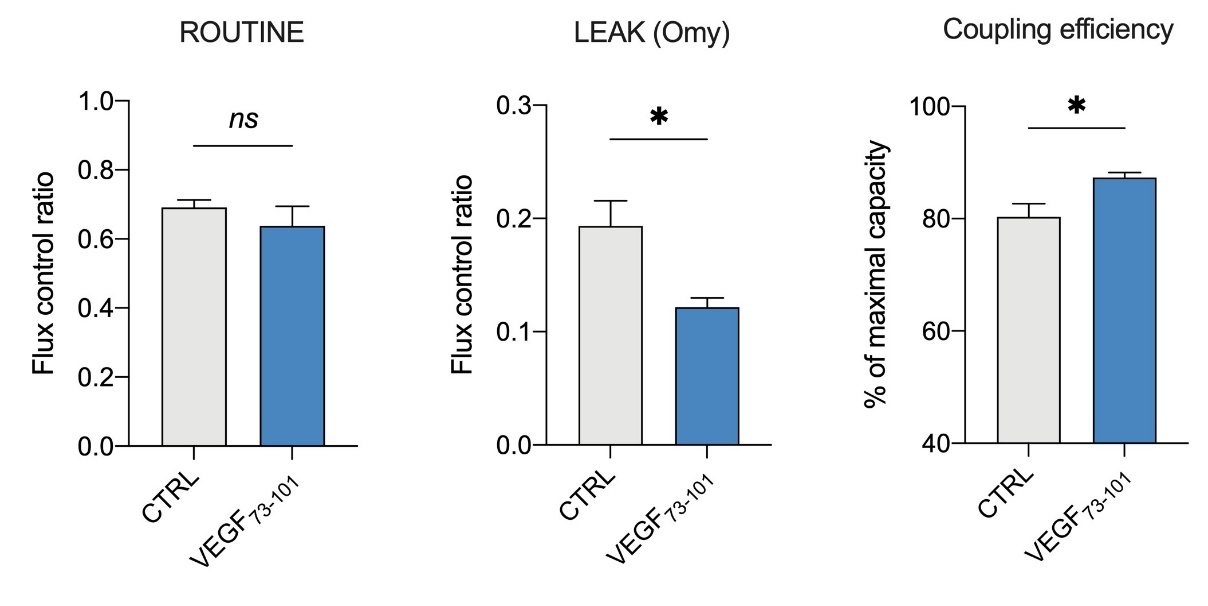
Suppl. Fig. 2**

**Supplementary Figure 2** Analysis of the respiratory profile of intact cells exposed to VEGF_73-101_ peptide. The contribution of ROUTINE and LEAK state to the maximal ET capacity, and the coupling efficiency in the ET state are shown as FCRs or as a percentage of the relative state in cells exposed for 24 h to 5 µM VEGF73-101 peptide. Untreated cells were used as control. Data are expressed as means ± SEM of n=3 independent experiments with n=2 each, and analyzed by unpaired t-test with *p<0.05; ns, not significant.

**Suppl. Fig. 3**

**
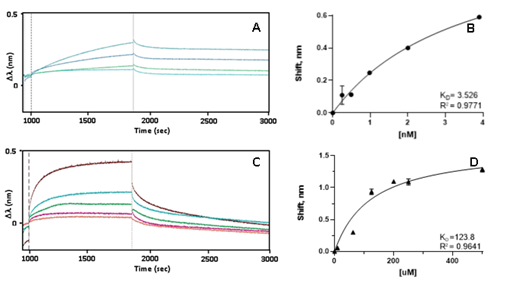
**

**Supplementary Figure 3** BLI analysis of the interaction between VEGFR-2/VEGF and VEGFR-2/VEGF_73-101_. VEGFR-2 binding to VEGF or peptide fragment was measured in a concentration range of 0.075-2 nM and 0.5-500 mM, respectively, for 900 sec in tube. Dissociation was followed for 1200 sec. The wavelength shifts recorded at 900 sec (at steady-state point) after the start of binding were plotted against the corresponding complex concentration in order to calculate the respective K_D_ values. Data were fitted to the non-linear equation, one site-Specific binding: Y=Bmax*X/(K_D_+ X), where Bmax is the maximum binding in wavelength shift and X is the concentration of analyte. The coefficients of determination (R^2^) and K_D_ values obtained are shown in the graphs for each complex **(A)** Binding sensorgram of VEGFR-2 (25 nM) with VEGF (2 nM, 1 nM, 0.5 nM, 0.075 nM). **(B)** Binding affinity curve between VEGFR-2 and VEGF_73-101_. **(C)** Binding sensorgram of VEGFR-2 (25 nM) with VEGF_73-101_ (500 µM, 250 µM, 125 µM, 10 µM, 0.5 µM). **(D)** Binding affinity curve between VEGFR-2 and VEGF_73-101_. Data are expressed as means ± SEM of n=2 independent experiments.

**
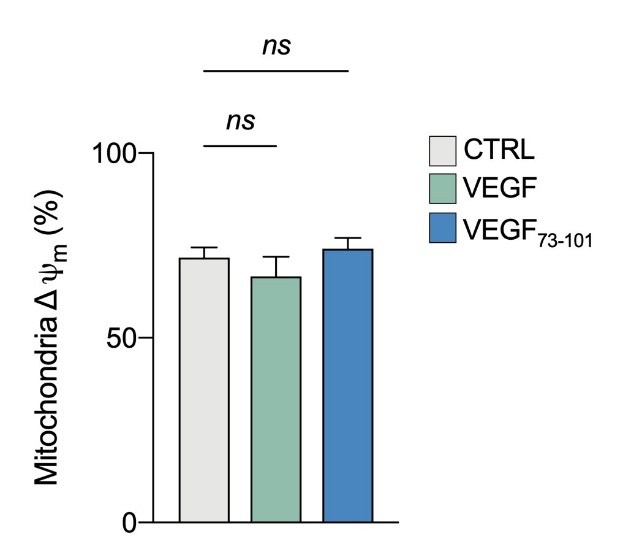
Suppl. Fig. 4**

**Supplementary Figure 4** Analysis of mitochondrial membrane potential of differentiated SH-SY5Y cells treated or not with VEGF or VEGF_73-101_ peptide for 24 h. Data are expressed as means ± SEM of n=4/5 independent experiments performed in duplicate and analyzed by one-way ANOVA followed by Dunnett’s test; ns, not significant.


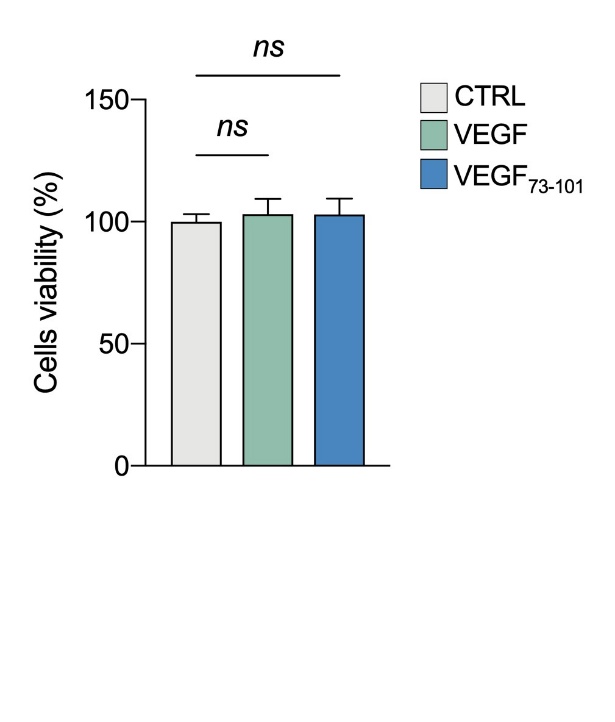
**Suppl. Fig. 5**

**Supplementary Figure 5** Analysis of cell viability by MTT assay of differentiated SH-SY5Y cells treated or not with VEGF or VEGF_73-101_ peptide for 24 h. Data are expressed as means ± SEM of n=3 independent experiments each performed at least in triplicate and analyzed by one-way ANOVA followed by Dunnett’s test; ns, not significant.


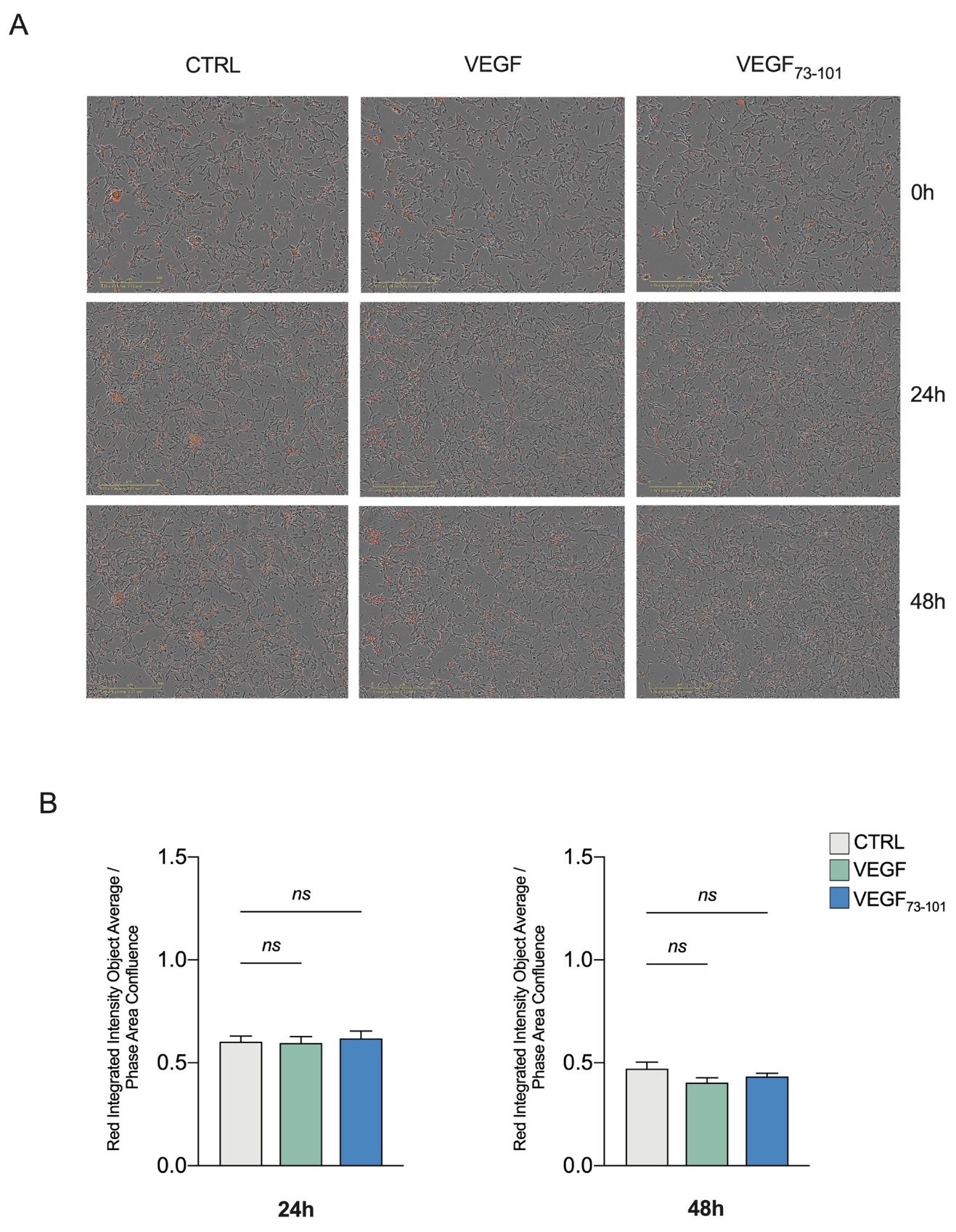
**Suppl. Fig 6**

**Supplementary Figure 6** Cytotoxicity assay of differentiated SH-SY5Y cells treated with 10 ng/ml (0.26 nM) of VEGF or 5 µM of VEGF_73-101_ peptide for 24 h or 48 h. Incucyte images showing Cytotox Red-positive cells taken at 0, 24 and 48 h of run time at 10x objective **(A)**. Quantification of death cells after 24 h and 48 h treatment (B)**.** Results are expressed as the ratio of Red Integrated Intensity (Objective Average) to the Phase Area Confluence, normalized to 0d0h0m. Data represent the mean of ± SEM of triplicates, with untreated cells as negative control. Data were analyzed by one-way ANOVA followed by Dunnett’s test. ns, not significant.
