# Supplementary material for: VEGF and the VEGF73-101 Fragment Prevent MPP+ Induced Mitochondrial Dysfunction in a Cell Model of Parkinson’s Disease
Source: Mol Neurobiol. 2025 Jul 31;62(12):16045–60. doi: 10.1007/s12035-025-05213-9 (PMC12559092; doi:10.1007/s12035-025-05213-9)
Supplement: Supplementary file 2 — (PDF 362 KB) [file 12035_2025_5213_MOESM2_ESM.pdf]

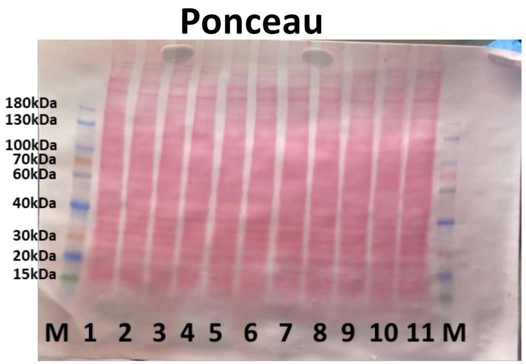

M – marker
1. CTRL
2. VEGF-A
3. Peptide73-101
4. CTRL
5. CTRL
6. Peptide73-101
7. VEGF-A
8. CTRL
9. VEGF-A
10. Peptide73-101
11. CTRL
M - marker

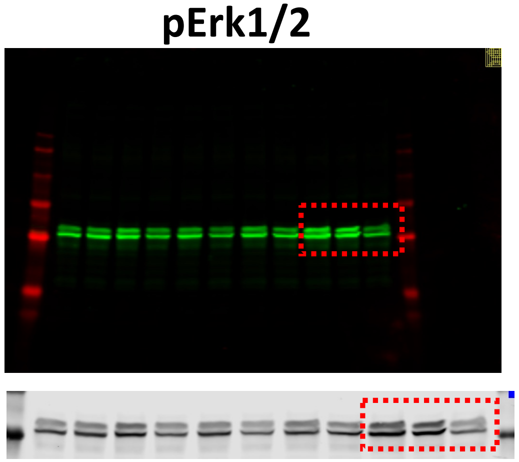

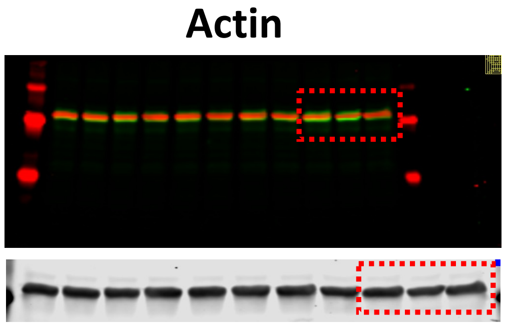

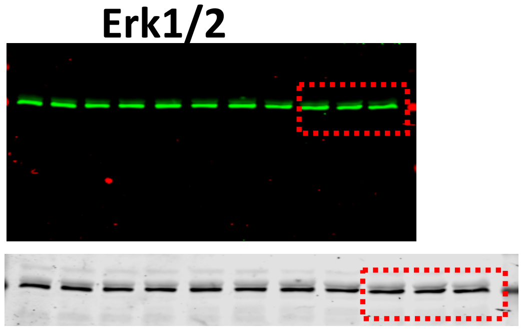

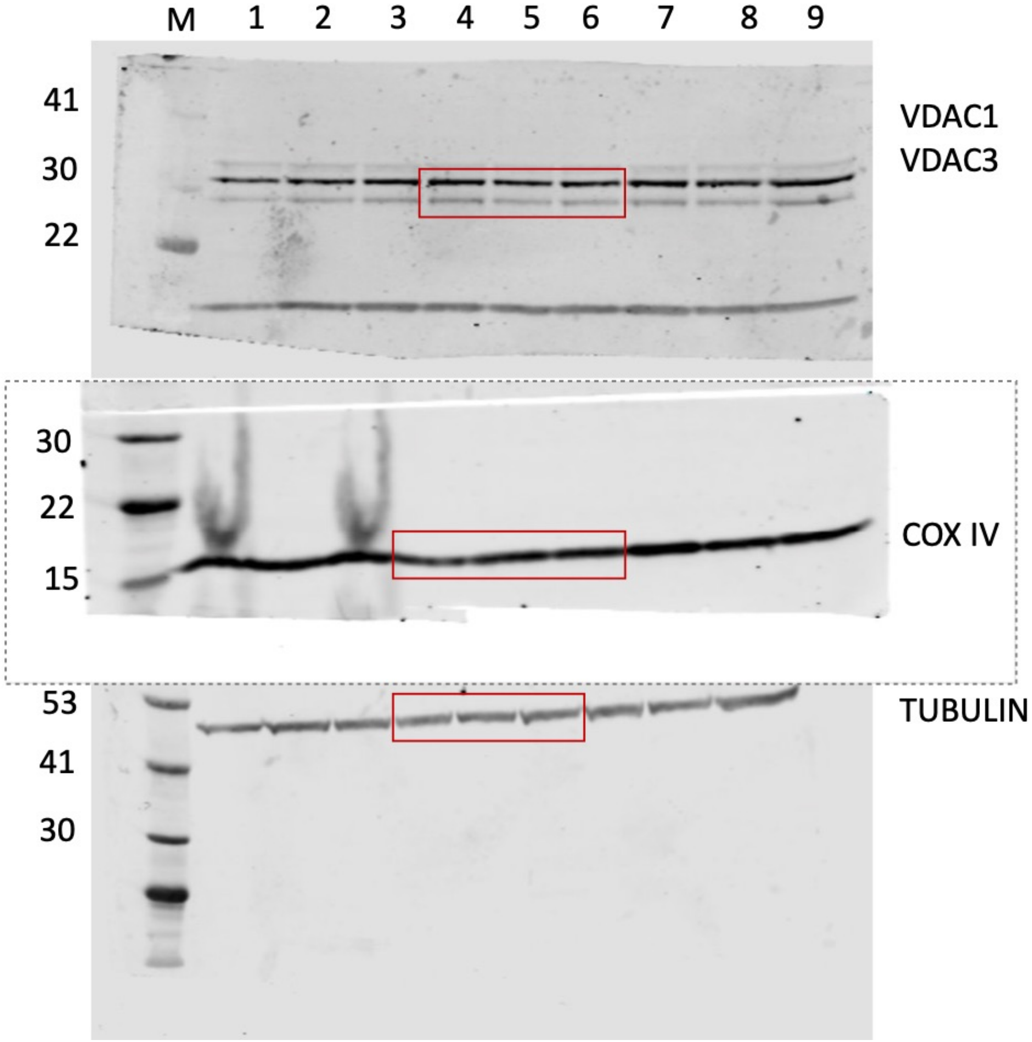

M Marker
1. CTRL
2. VEGF
3. Peptide73-101
4. CTRL
5. VEGF
6. Peptide73-101
7. CTRL
8. VEGF
9. Peptide73-101
